# Amalgamation and Optimization of FAEE Biodiesel Fuel from *Croton macrostachyus hochst. ex Delile* Seed oil Utilizing Solid Calcium Oxide-Chicken Eggshell Squander as a Heterogeneous Catalyst

**DOI:** 10.1155/2022/7984077

**Published:** 2022-12-10

**Authors:** Yigrem Solomon, Ermias Girma, Ancha Venkata Ramayya

**Affiliations:** ^1^Department of Mechanical Engineering, Wollega University, Nekemte, Ethiopia; ^2^School of Chemical Engineering, Jimma Institute of Technology, Jimma University, Jimma, Ethiopia; ^3^Faculty of Mechanical Engineering, Jimma Institute of Technology, Jimma University, Jimma, Ethiopia

## Abstract

Identification of better yielding nonedible feedstocks and process improvements with locally prepared chemicals is the way forward for improving biodiesel economic viability. Synthesis of fatty acid ethyl ester (FAEE) biodiesel from *Croton macrostachyus hochst. ex delile* (*C. M.*) seed oil is investigated through the transesterification method including oil extraction and CaO-chicken eggshell waste as a heterogeneous catalyst and ethanol, which has not yet been investigated earlier. The effects of catalyst load from 2% to 6% (w/w) of the weight of oil, ethanol to oil ratio from 9 : 1 to 12 : 1(v/v), reaction temperature from 60 to 80℃, and reaction time variation from 0.45 to 2 hr have been explored. The yield has been investigated, with the oil content of the *C. M.* seed being 46.85% realized through the solvent extraction route. The central composite design (CCD) using a design expert is employed to investigate the effects of different process parameters for biodiesel synthesis and to find the optimum conditions for maximizing the yield. The conversion to biodiesel via the calcium oxide-heterogeneous catalyzed transesterification route has achieved 91% yield under the optimum conditions. The optimum result predicted by the model is found to be 91.036% at a catalyst loading of 5.802 wt.%, reaction temperature of 76.117℃, a reaction time of 1.969 hr, and ethanol to oil molar ratio of 11.55 with a desirability value of 1.000. The FTIR spectrum confirms the composition and functional groups of synthesized biodiesel under optimum reaction conditions. Physicochemical properties of synthesized *C. M.* biodiesel are determined, and the results when compared with the biodiesel standard specifications are within the range prescribed. The result showed that *C. M.* could be a very viable feedstock for the biodiesel industry, which could be exploited as an alternative fuel source.

## 1. Introduction

Design for sustainability in the present context calls for decarbonization, distributed generation/production, and dematerialization, i.e., conservation of depletable materials. Biodiesel production from indigenous nonedible feedstocks holds promise to partially substitute a more significant share of fossil-based resources and mitigate the associated GHG emissions. However, to improve the economic viability of biodiesel, ways and means are to be explored, such as using locally available materials and alcohol including catalysts. The potential of *Croton megalocarpus* [1] and *Croton macrostachyus* (*C. M.*) [2] have been explored to synthesize biodiesel fuel, and attributes like higher yield per hectare compared to Jatropha as well as the attractive cold flow properties of Biodiesel formed from these feedstocks have been highlighted. While it has been reported that the oil content of the seeds from both species is roughly the same, strategies intended to remunerate ecological execution and supportability of biofuels while further developing the transformation cycle to energize the arrangement of the more abundant and geologically broader feedstock supply could see second-generation items start to obscure the first-generation in the medium- to longer-term [2, 3]. *C. M.* is one of the best raw materials for producing biodiesel as it is nonedible, occurs throughout tropical Africa and is one of the native trees in Ethiopia.

### 1.1. The *Croton macrostachyus hochst ex. delile* Description

**Figure 1:**
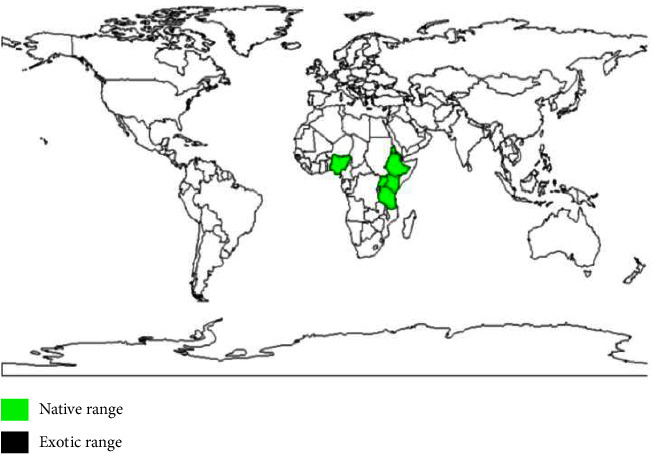
*Croton macrostachyus hochst ex. delile* species geographical distribution [Orwa et al. [20]].

Monoicous or dioicous, deciduous, medium-sized tree up to 25–30 m tall; bole cylindrical, up to 100 cm in diameter; bark grey to grey-brown, inner bark pale brown to reddish-brown, smell peppery; crown rounded and open with large, spreading branches; young branches densely stellate hairy. Leaves substitute, straightforward, becoming orange prior to falling; stipules direct, up to 15 mm long, before long falling; petiole up to 12–20 cm long, with 2 stalked glands at the top; sharp edge applauds curved to practically round, up to 17–25 cm × 14–20 cm, base cordate, summit taper, edges sporadically toothed, thickly stellate shaggy on the two sides, whitish-green underneath [4, 5]. *Croton macrostachyus hochst ex. delile*, known as broad-leaved croton in English, is named by various vernacular names in Ethiopia's different locations, Bakkanniisa/Makkaniisa (Afaan oromoo), Bissana (Amharic), Masincho (Sidama), and Ambuk (Tigrigna) [2, 6]. The geographical distribution is shown in Figure 1.

Nonedible oil such as *C. M.* oil can become a handy alternative raw material to produce biodiesel as a result of the massive demand for edible oils and also on account of food versus fuel competition.

**Figure 2:**
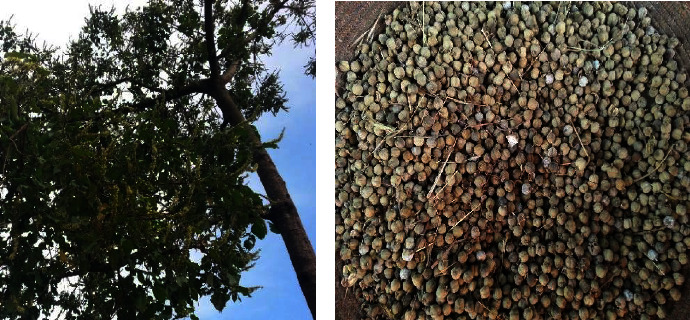
*C. M hochst. ex delile* (a) plant, (b) seed (own photo).

As a result of its drought resistance and faster growth, a *C. M.,* shown in Figure 2 as a plant and its seed, stays well-thought-out beneficial species used for changing water stream banks and tainted wasteland. Besides, due to the oil content of the seed and availability of feedstock for the synthesis of biodiesel, it can be a candidate for alternative fuel plants.

Several possible nonedible oil feedstocks were studied to synthesize biodiesel fuel in this backdrop, such as the castor and jatropha seed. In contrast, previous research based on *C. M*. seed oil has employed methanol and a homogeneous catalyst [1, 2]. Despite the fact that the homogeneous base-catalyzed biodiesel creation processes are moderately quick and show high return, they are as yet not extremely cost-effective with petrodiesel ones due to the expense of unrefined substance utilized impetus that cannot be recuperated after the response [1, 3]. Nevertheless, using a heterogeneous catalyst (CaO) offers many benefits, for example, higher reaction, gentle response conditions, reusability, simple accessibility, and minimal expense [7–11]. Besides, previous studies have employed methanol as alcohol for biodiesel production, which is expensive and toxic relative to ethanol, now widely being produced from renewable sources in sugar-based distilleries, unlike the case of methanol. Large-scale cultivation, production, and related lipid recuperation are bound by various variables, particularly microalgae inferred biodiesel are seen as expected feedstocks for biofuel creation. The economic feasibility of biodiesel yield is mainly affected by feedstock cost. Acid catalysts are cost-effective to produce biodiesel from the affordable feedstock. Cheap and useable catalysts reduce production costs and ameliorate productivity [4, 5]. Supplanting petroleum derivative energies with biofuels, such as biodiesel created from sustainable natural material has the potential to decrease a few bothersome aspects of nonrenewable energy usage, including customary and ozone-harming substance poison outflows, modest asset decrease, and reliance on shaky unfamiliar providers. Interest in biofuels could likewise increase ranch pay. Then again, in light of the fact that various biofuel feedstocks need land, water, and different assets, previous research suggests that biodiesel might bring about a few unwanted impacts [3]. Biodiesel synthesis includes two fundamental subprocesses specifically oil extraction [12, 13] cum cleansing [8, 14] and transesterification [15]. The yield of biodiesel produced through transesterification is impacted by a few interaction factors [16]. The response is either inadequate or the yield is diminished significantly if the parameters are not optimized. The decontamination interaction is the wet washing technique, which utilizes water or fermented water to refine the esters and dry washing, which also utilizes strong adsorbents or particle exchange pitches to purge unrefined biodiesel [9, 10]. Furthermore, the RSM approach can be utilized to advance the biodiesel interaction parameters by soluble transesterification. While a few investigations revealed the improvement of biodiesel creation utilizing the RSM procedure, the streamlined utilization of RSM coordinated with the allure capability approach is ineffectively known [11].

Biodiesel can't as of now be considered a serious contender to diesel fuel owing to the enormous distinction in their worldwide creation, with a high volume. It is not adequate for biodiesel to meet entire needs and necessities; it should likewise battle financially with fossil diesel at large-scale creation [12]. According to the previous studies [17–19], the main factors influencing biodiesel yield after transesterification are reaction temperature, alcohol to oil molar ratio, time of reaction, mixing intensity, and amount of catalyst loaded. Hence, in the present study, the production of biodiesel through the reaction of the *C. M.* seed oil with ethanol within the sight of a heterogeneous catalyst, CaO is investigated, and optimum biodiesel conversion is analyzed at the ideal mix of process variables such as ethanol to oil proportion, amount of catalyst loaded, time of reaction, and reaction temperature. The properties of *C. M.* biodiesel are also tested similarly, contrasted, and suggested along with ASTM and EN standards. Reusability consideration led to the selection of chicken eggshell leftover as a heterogeneous catalyst to make the biodiesel process more economically viable and environmentally friendly.

## 2. Materials and Method

### 2.1. Site Description

The *C. M.* seed samples used for the synthesis of Biodiesel are collected from Jimma, in the Oromia region, the largest city located in the southwestern part of Ethiopia. It has a latitude of 7°40′12″N and longitude of 36°49′48″E, with a height of 1780 m above sea level, which is appropriate for the growth of *C. M.* plants.

### 2.2. Materials and Equipment

The significant materials and equipment used during the experimental work are *C. M.* seed, waste chicken eggshell, ethanol, oven, grinder, measuring cylinder, muffle furnace, Soxhlet apparatus, rotary evaporator, bomb calorimeter, separating funnel, distilled water, phenolphthalein, water bath, heating mantle, digital balances, filter paper, bunsen burner, sodium hydroxide, potassium hydroxide beakers, water bath, and different sizes of conical flasks. All the other chemical compounds utilized are of analytical reagent grade.

### 2.3. *C. M.* Seeds Sample Preparation

**Figure 3:**
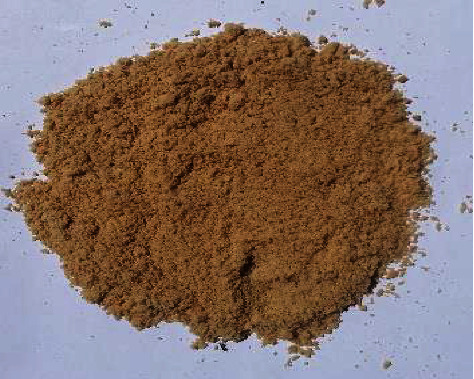
Crushed *C. M.* seed.

Seeds were crushed to rupture the cell wall and release the solute; see Figure 3. for direct contact with the solvent during the contact equilibrium process.

### 2.4. Extraction of *C. M.* Seed Oil

The dried seeds are squashed in a crushing mill with a constituent part size of less than 2.0 mm. Then, the crushed *C. M.* seeds are packed into thimble and n-hexane of analytical grade as the solvent is set in the extraction unit at a dissolvable-to-the-solid ratio of 5 : 1 (v/w). Solvent to solid proportion is picked by their utilization according to their convention possibility in industrial scales and based on previously reported results and patent data [20].

### 2.5. Chicken Eggshell Waste Catalyst Preparation

**Figure 4:**
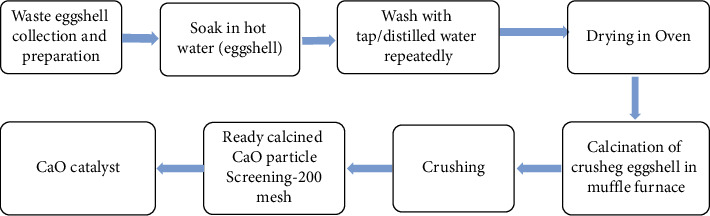
Block diagram showing the preparation of catalyst CaO-eggshell waste-derived process (own figure).

The eggshell is washed using refined water to eliminate impurities and afterward dried in an oven (105°C, 24 h). The sample is squashed in agate mortar hardware into fine particles (0.25–2 mm). The fine particles are then calcined in a muffle furnace at a temperature of 900°C for 2 hours to dispense any type of carbon and get the total transformation from CaCO_3_ to CaO. The debris then is utilized as a heterogeneous base catalyst in the development of ethyl ester. The whole sequential process is highlighted in Figure 4.

#### 2.5.1. Catalyst Characterization

XRD is one of the most commonly used techniques for either crystalline or amorphous phase identification. The samples are investigated in a Shimadzu diffractometer XRD Y3000 (Shimadzu, Nakagyo-Ku, Kyoto, Japan), using Cu-Ka radiation (*λ* = 1.5406 Å), functioning at 30 kV and 25 mA and by a scanning speed of 0.03°/s.

### 2.6. Experimental Work Design

During this study, four process variables that distress the synthesis of Biodiesel, such as the reaction temperature, catalyst loaded, ethanol-to-oil proportion, and reaction time have been varied; twenty-six experiments are conducted. Then, the data are statistically investigated by the design-expert 11.1.2 software. A three-level-four-factor Table 1 and two center points are used with a central composite design (CCD). The CCD was applied for carrying out the optimization studies to maximize the yield of biodiesel in the transesterification method.

Experimental values of converting the *C. M.* seed oil to biodiesel at the design points at different parameters are obtained, and from those values, the yield is determined and recorded.

(1) $$\begin{array}{c} Biodiesel\;yield\left(\%\right)=\frac{weight\;of\;biodiesel\;obtained\left(gm\right)}{weight\;of\;oil\;used\left(gm\right)}\times 100\%\text{.} \end{array}$$

## 3. Results and Discussion

### 3.1. Seed Preparation

The collected seeds are separated from the chaff and other impurities and then weighed before drying to remove the excess moisture. The moisture content for the three samples characterized is 7.366%, 5.425%, and 4.22%, respectively, and the average moisture content is obtained as 5.67% w/w, which is in the acceptable range for oil extraction [21].

### 3.2. Oil Extraction

Extraction of *C. M.* seed oil is carried out using heating and solvent extraction methods. From 100 g of purified seed, 19.8 g oil is found, which shows 19.8% (w/w) of *C. M.* seed oil is extracted using heating methods while from 100 g of purified seed, 46.85 g oil is found, which shows 46.85%(w/w) of *C. M*. seed oil is extracted using solvent extraction. The properties of the seed oil in comparison with others are indicated in Table 2.

### 3.3. Physicochemical Properties of the Chicken Eggshell Ash

**Figure 5:**
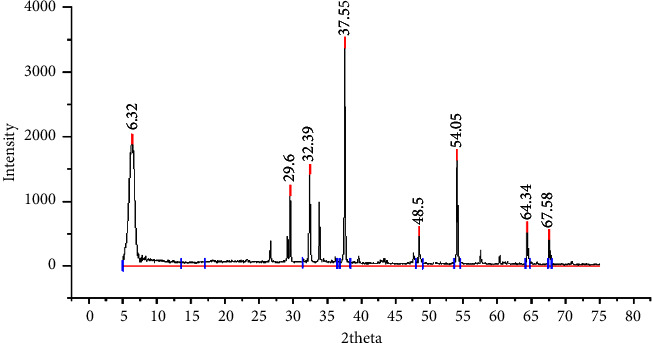
The XRD form of calcium oxide powder calcined at a temperature of 900°C (own figure).

This study inspected the chemical composition of waste chicken eggshells before and after calcination via XRD investigation.  Figure 5 shows the chemical structure before and after calcination; the eggshells became totally white for all intents, which shows that calcium carbonate got away and the item just became calcium oxide. The particle size of calcium oxide calcinated at 900℃ is almost 36.4 nm, and the intense sharp peaks observed at 2*θ* are 6.32°, 29.6°, 32.39°, 37.55°, 54.05°, 64.34°, and 67.58°^,^ which corresponds to the characteristics of CaO. XRD spectra of calcined eggshell tests are acquired with a cu-ka (*λ* = 0.15405 nm) at 37.55°, a scan speed of 0.030°/second, and a scan range from 5° to 75°, which shows the decomposition from CaCO_3_ to CaO. The pinnacle points are like those reported in past studies [22, 23].

### 3.4. Factors Influencing the Yield of Biodiesel Production

#### 3.4.1. The Effect of Ethanol to Oil Ratio

The highest biodiesel yield is obtained at the molar ratio of 10.5 : 1, while it decreased after the molar ratio of 10.5 : 1. This is on the grounds that by expanding ethanol content, glycerin is generally disintegrated in abundance ethanol, holding ethanol back from responding with the catalyst and subsequently makes it challenging to isolate ethanol from biodiesel and glycerin [25].

#### 3.4.2. The Effect of Response Temperature

As the temperature increases from 65 to 75℃, a significant increment in biodiesel yield is observed. An increase in the yield of FAEE at the higher response temperature is because of the higher reaction rate since transesterification is fundamentally diffusion controlled. However, as the temperature approaches the boiling point of ethanol, the percentage yield of FAEE decreases. From this experiment, the increase of reaction temperature clearly shows how Biodiesel yield and the maximum yield of biodiesel are obtained at 70℃. Likewise, an increase in the temperature strengthens the saponification reaction of the fatty oil [26].

#### 3.4.3. The Effect of Reaction Time

The maximum biodiesel yield is obtained at the elapsed time of 1.375 h. Gradually, the saponification phenomenon is seen over some time. As such, exorbitant reaction time diminishes biodiesel yield because of the regressive reaction. Hence, the reaction time is a restricting variable on biodiesel production yield and can influence biodiesel synthesis if it exceeds the maximum value [27, 28].

#### 3.4.4. The Effect of the Catalyst Load

Catalyst load is seen to influence the biodiesel yield up to a specific concentration positively. Beyond this concentration, the biodiesel yield diminished with an increase in CaO concentration. This can be attributed to the fact that the amount of available catalytic active surface is crucial for shifting the reaction equilibrium forward and ethanol in the reaction mixture [29].

### 3.5. Statistical Investigation of the Experimental Results

The statistical investigation of the model has been performed to assess the analysis of variance (ANOVA). It is utilized to produce surface plots, utilizing the fitted equation obtained from the regression analysis, holding one of the autonomous variables as constant. The central composite design (CCD) predicts values and variables that lead to accomplishing a high yield of biodiesel in actual terms. The transesterification process response is used to develop a numerical model that correlates the yield of FAEE to the transesterification interaction factors investigated. The Design-Expert version 11 software (Stat-Ease Inc., Minneapolis, USA) has been utilized for the regression study of the experimental data and valuation of the statistical significance of the equation developed.

The typical model equation that links the response (% yield of FAEE) to the transesterification process factors in terms of the coded factors is given by the following equation:

(2) $$\begin{array}{c} FAEE\;yield\left(\%\right)=86.7+2.83\times A+1.15\times B+3.03\times C+2.20\times D+0.8438\times AB+1.66\times AC-1.76\times AD-2.14\times BC-7.74\times A^{2}-5.44\times B^{2}+1.96\times C^{2}+2.21\times D^{2}\text{,} \end{array}$$

where, *A*-a reaction temperature, *B*-ethanol to oil ratio, *C*-reaction time, and *D*-catalyst load. *A.B.*, *A.C.*, *A.D.*, and *B.C.* are interaction effects.

#### 3.5.1. The Effect of Interaction of Process Variables on Percentage Yield of FAEE

**Figure 6:**
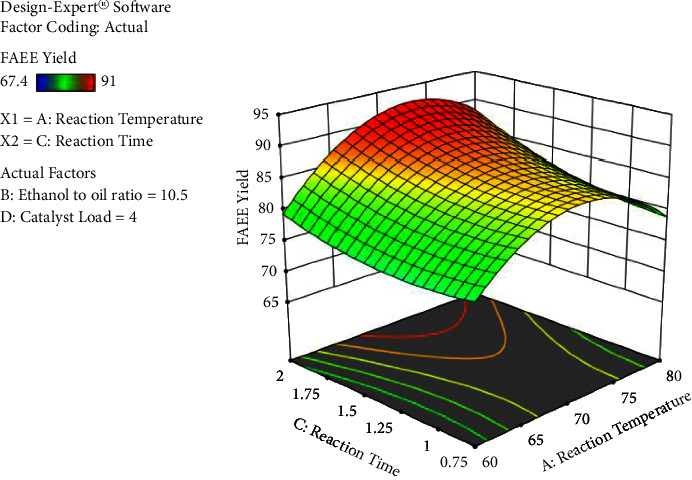
The interaction effect of reaction temperature and reaction time versus percentage yield of FAEE (own figure).

**Figure 7:**
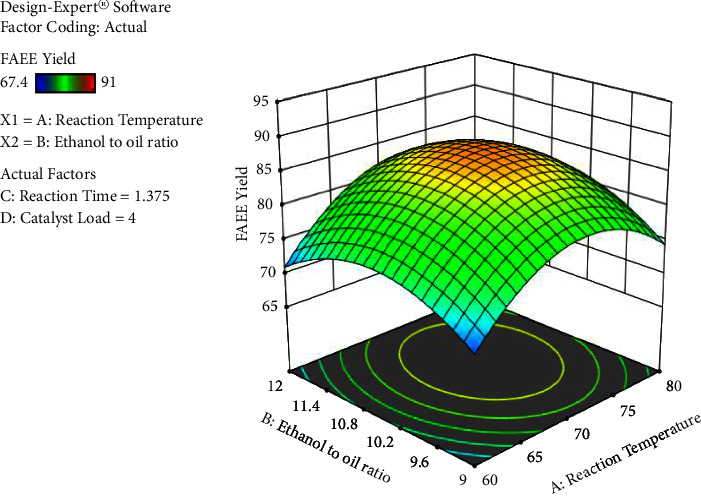
The interaction impact of the reaction temperature and ethanol to oil ratio versus percentage yield of FAEE (own figure).

**Figure 8:**
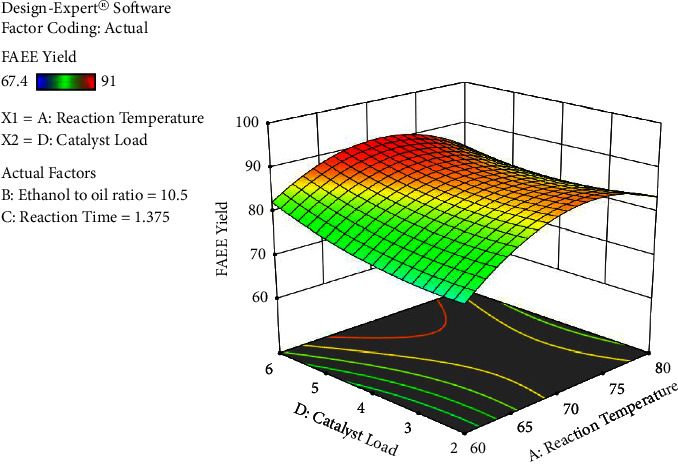
The interaction impact of the reaction temperature and catalyst load versus yield of FAEE (%) (own figure).

**Figure 9:**
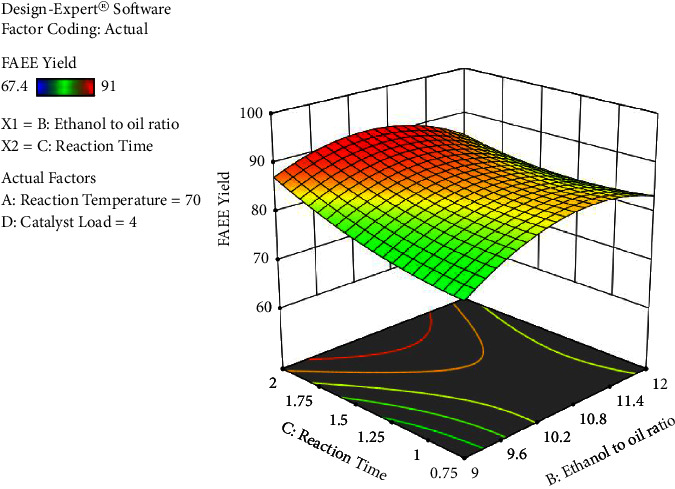
The interaction impact of ethanol to oil ratio and reaction time versus percentage yield of FAEE (own figure).

The process factors have significant interaction effects except for ethanol's molar ratio to oil with catalyst load interaction. The interaction result between ethanol to oil ratio and reaction time has a significant (*p* < 0.0001) result on the biodiesel yield, followed by response temperature and catalyst loading, the reaction temperature and reaction time, reaction temperature, and ethanol to oil proportion in that order, respectively. The results obtained are presented in Figures 6–9.

As observed from Figure 6, the reaction temperature and reaction time have small interactions prior to 70°C and they have positive effects on the yield of biodiesel at 4% catalyst loading until the ethanol to oil ratio reaches 10.5 : 1. But, beyond 10.5 : 1 ethanol to oil ratio and at fixed catalyst loading, the yield of biodiesel slightly decreases due to the fact that emulsion is formed.

Figure 7 shows the effect of reaction temperature and ethanol to oil ratio on biodiesel yield when the catalyst concentration is 4%. As the temperature of the reaction is increased, at lower oil to molar ratio the yield of biodiesel is also increased. The biodiesel yield also increases as the ethanol to oil ratio increases up to an optimal level and decreases thereafter.

As can be inferred from Figure 8, upon increasing the catalyst loading from 2 to 6% with an increase of reaction temperature from 60 to 80°C, the yield of biodiesel increased substantially. Beyond this ratio, the yield of biodiesel seems to gradually decrease. The highest yield was obtained at an ethanol to oil ratio of 10.5 : 1, reaction time of 1.375 hr, and at a reaction temperature of 70°C with 4% catalyst loading.

As indicated in Figure 9, there is an interaction effect between reaction time and ethanol to oil ratio. It can be seen that with the reaction time rising, the FAEE yield generally decreases. The reason is that as the reaction time increases ethanol to oil ratio tends to decrease on account of conversion.

### 3.6. Optimization of Interaction Variables

The enhancement of process factors inside the chosen ranges for the maximum change from the oil to FAEE is carried out. The optimum outcome predicted by the model is found as 91.036% at a catalyst loading of 5.802 wt.%, reaction temperature of 76.117℃, a reaction time of 1.969 hr, and ethanol to oil molar ratio of 11.55 with a desirability value of 1.000. The improved optimization result also reveals a similar outcome as the ANOVA output. The predicted (91.036%) and experimental output (91%) values of (biodiesel yield) under the optimum circumstances are in excellent agreement. In other cases, the minimal deviation, i.e., 0.036%, among the predicted and experimental values of % (biodiesel yield) indicates that the typical equation is suitable and adequate to forecast the biodiesel yield from the process using *C. M.* seed oil, ethanol, and CaO-chicken eggshell waste derived as a heterogeneous catalyst via the transesterification reaction process in the range of variables investigated. A comparison of the *C. M.* seed oil-based biodiesel properties from this study is presented in Table 3 against those of WCO biodiesel.

### 3.7. Analysis of FTIR for *C. M.* Derived Biodiesel Characterization

**Figure 10:**
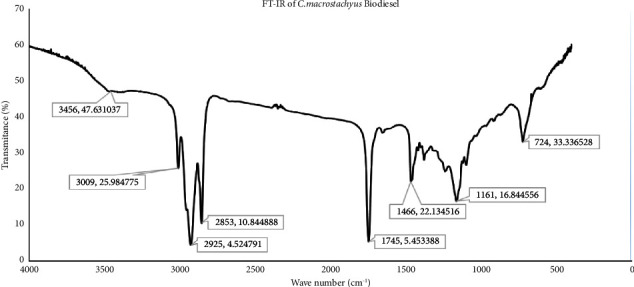
The FTIR obtained for *C. M*. based biodiesel produced in this study.

FTIR has been carried out to characterize the biodiesel produced in this study from *C. M.* seed oil. The spectrum obtained are shown and analyzed in Figure 10.

The broad absorption band at 3456 cm^−1^ in above Figure 10 is accredited to the *O. H.* stretch vibrations group of the water molecule. Sharp bands at 2925 and 2853 cm^−1^ are attributable to the C-H stretch vibrations of the ethylene groups. The firm peaks at 1745 cm^−1^(C=O ester) and 1161 cm^−1^(C-O ester) that are available inside the range are allotted to carbonyl functional groups; these two functional groups affirm the presence of ethyl esters and are accredited to biodiesel, and this perception is in accordance with the experimental data reported in [17, 31, 32]. The observed dominant peaks represent the triglyceride functional group, which is the major component of the *C. M*. biodiesel. Besides, comparing the properties of biodiesel produced in this study with those specified by international standards is illustrated in Table 4. As perceived in the table, most of the physicochemical properties of the *C. M.* seed oil biodiesel are in good agreement with international standard prescriptions.

## 4. Conclusion

In this study, the synthesis and optimization of biodiesel from *C. M*. seed oil using calcium oxide-chicken eggshell waste as a heterogeneous catalyst are investigated. The experimental oil yield obtained from washed and dried *C. M.* seed oil using the solvent extraction route is 46.85% (w/w), whereas using the heating method is 19.8% (w/w). Degumming is seen to be sufficient to reduce the FFA level. The biodiesel extracted using the transesterification method is characterized, and the yield from the experiments conducted has been examined by employing the design-expert software for optimizing the process yield. The process variables revealed a significant interaction effect on the FAME yield based on the experimental results obtained. Reaction time and reaction temperature have a more profound effect on the FAEE yield than other process variables such as catalyst loading and ethanol to oil ratio. The optimum result predicted by the model is found to be 91.036% at a catalyst load of 5.802 Wt.%, reaction temperature of 76.117℃, a reaction time of 1.969 hr, and ethanol to oil molar ratio of 11.55 with a desirability value of 1.000. The optimization outcome also reveals the same result as the ANOVA output. The predicted (91.036%) and experimental (91%) values of biodiesel yield under the optimum conditions are in excellent agreement. The minimal deviation, i.e., 0.036%, between the predicted and experimental values of the percentage of biodiesel yield indicates that the model equation is appropriate and more than adequate to predict the biodiesel yield process using *C. M.* seed oil, ethanol, and CaO-chicken eggshell waste as a heterogeneous catalyst via transesterification reaction in the range of variables investigated. The FTIR spectrum result confirms the presence of biodiesel. The physicochemical properties of synthesized *C. M.* biodiesel determined are observed to be within the range allowed by ASTM and EN standard values.

Considering the compatibility with the standards, *C. M.* seed can be an alternative feedstock for the synthesis of biodiesel to help the country in substituting fossil fuel imports in the form of blended fuels. Keeping in view all these properties, mega cultivation of this crop may be carried out in the future to synthesize biodiesel at a larger scale that could have positive impacts and will generate new jobs for the poorest of local communities.

## Figures and Tables

**Table 1:** Experimental work design for four factors.

Process variables	Symbols	Lower limit	Higher limit
Reaction temperature (℃)	A	60	80
Ethanol to oil ratio (v/v)	B	9	12
Reaction time (hr.)	C	0.75	2
Catalyst load (wt.%)	D	2	6

**Table 2:** Summary of physicochemical properties of *C. M.* seed oil with another feedstock [22].

No.	Physicochemical property	Units	*C. M*. seed oil (present study)	^∗^Waste cooking oil	^∗∗^Castor oil	^∗∗∗^Jatropha curcas oil
1	Density	kg/m^3^	892	900	890	907
2	Kinematic viscosity	mm^2^/s	42.37	49.93	40.39	49.93
3	Acid value	mgKOH/g	2.36	2.4	3.4	3.38
4	Free fatty acid	%	1.18	1.2	1.7	1.7
	C.M. seed oil	%	46.85	—	35–40	35–40
5	Saponification value	mgKOH/g	198.03	199	198.63	193.55
6	Moisture content	%	1.68	—	—	
7	Ash content	%	0.73	—	—	
8	Iodine value	gI_2_/100 g	64.0	73	65	20.3–93.9

^∗∗∗^Adopted from [19]. ^∗∗^Adopted from [23]. ^∗^Adopted from [24].

**Table 3:** Summary of physicochemical properties of C.M. seed oil-based biodiesel with standards [30].

Property	*C. M*. biodiesel (this study)	Unit	^∗^WCO biodiesel	ASTMD 6751	Test methods
Density at 20°C	878	Kg/m^3^	885	875–900	ASTMD 4052
Moisture content	0.047	%		<0.05	EN ISO12937
Kinematic viscosity at 40°C	3.54	mm^2^/s	4	1.9–6	ASTM D 445
Acid value	0.87	mgKOH/g oil	0.62	≤0.9	ASTM D664
FFA composition	0.435	%		—	ASTM D664
PH	7.83	—	8	7–9	—
Saponification number	120	mgKOH/g oil	200	190–230	ASTMD5558
Calorific value	40.2	MJ/kg	41	40–42	ASTM D 240
Iodine value	102.1	gI_2_/100g	103.8	—	EN 14111
Flashpoint	198	℃	160	130	ASTM D 93
Ash content	0.023	%		0.02	ASTM D 482
Cetane number	56	—	56	≥47	ASTM D976

**Table 4:** Correlation of *C. M.* biodiesel with standards.

Physicochemical properties	Units	*C. M*. biodiesel (present study)	^∗^ASTM D 6751–10	^∗^EN	^∗^Diesel fuel	Test method
				14214		
Density at 20 ℃	kg/m^3^	878	875–900	860–900	840–860	(EN ISO3675 &ASTMD445)
Moisture content	% w/w	0.027	<0.03	—		EN ISO12937
Kinematic viscosity at 40 ℃	mm^2^/s	4.87	1.9–6.0	3.5–5.0	1.9–3.8	ASTM D 445
Acid value	mgKOH/g	0.48	≤0.8	≤0.5	—	ASTM D 664
Ash content	% w/w	0.021	<0.03	<0.02		ASTM D482
PH	—	7.83	7–9	—	5.5–8	—
Calorific value	M. J./kg	40.2	40–42	>35		ASTM D 240
Flashpoint	℃	198	≥ 130	≥ 120		ASTM D 93
Saponification value	mgKOH/g	120	≤215.99	≤218.79		ASTMD5558
Iodine value	g I_2_/100g	102.1	—	120 max	84	ASTMD 5558
Cetane number	—	56	≥ 47	≥ 51	47–55	ASTM D613

^∗^Adopted from [31].

## Data Availability

The data used to support the findings of this study are included within the article.
